# Skeletal Divergence and Condylar Asymmetry in Patients with Temporomandibular Disorders (TMD): A Retrospective Study

**DOI:** 10.1155/2021/8042910

**Published:** 2021-09-24

**Authors:** Maria Francesca Sfondrini, Letizia Bolognesi, Mario Bosco, Paola Gandini, Andrea Scribante

**Affiliations:** ^1^Unit of Orthodontics and Paediatric Dentistry-Section of Dentistry-Department of Clinical, Surgical, Diagnostic and Paediatric Sciences, University of Pavia, 27100 Pavia, Italy; ^2^Private Practice, Tortona, Italy

## Abstract

**Introduction:**

This study was aimed at evaluating the association between vertical skeletal patterns, condylar height symmetry, and temporomandibular disorders in adults.

**Methods:**

The study sample consisted of 200 patients (ages 18–30 years old) retrospectively recruited: 100 with temporomandibular disorders (TMD) and 100 without TMD (control), diagnosed by Diagnostic Criteria for the Temporomandibular Disorders (DC/TMD). For each subject, skeletal divergence was assessed on lateral cephalograms, and condylar height symmetry was evaluated by orthopantomography (Habets' method).

**Results:**

Subjects with temporomandibular disorders showed a strong association with condylar asymmetry (*p* < 0.0001) and, for the skeletal pattern variables, hyperdivergence (*p* < 0.001). A correlation with the female sex was also found (*p* < 0.04), while there was no difference in terms of age in the 2 groups (*p* > 0.29).

**Conclusions:**

Although it does not imply a direct cause-and-effect relationship, the present study suggests condylar asymmetry and hyperdivergent skeletal pattern are more likely to be associated with a higher risk of temporomandibular disorder joint diseases in adult patients.

## 1. Introduction

The most common objectives of orthodontic treatment are facial and dental aesthetics and the improvement in the masticatory function. Nowadays, the number of adults requiring orthodontic treatment is increasing [1]; for this reason, orthodontists must interface with a population where there is a high percentage of functional problems. Epidemiological studies show that approximately 33% of the population has at least one characteristic symptom and 56% has a clinical sign of temporomandibular disorders (TMD) [2, 3], and in particular, TMD symptoms have a broad prevalence peak between 20 and 40 years of age, with a lower prevalence in younger and older people [4, 5].

The aetiology of temporomandibular disorders is multifactorial, and numerous studies indicate factors such as traumas, stress, arthritic changes by systemic conditions, and parafunctional habits [6], but also, emotional/psychological aspects can influence the development or maintenance of TMD signs and symptoms [7–9]. Furthermore, craniofacial morphology has also been considered another factor related to TMD [10, 11]. For instance, a recent review has concluded that hyperdivergent jaw growth pattern and Class II skeletal profile are associated with a higher frequency of TMJ disc displacement and degenerative disorders [12].

In this context, main and routine examinations for the orthodontist, such as orthopantomography (OPG) and laterolateral teleradiography, could also be useful to obtain information from a functional point of view that can guide and be a wake-up call for the possible presence or potential development of TMD.

This study was aimed at evaluating the association between vertical skeletal patterns, condylar height symmetry, and temporomandibular disorders in adults.

The null hypothesis of the investigation was that there is no significant difference in terms of age, sex, craniomandibular divergence, and condylar symmetry between the two groups.

## 2. Material and Methods

This was a retrospective analysis of orthopantomograms and cephalometric radiographs of adult patients who were seeking orthodontic treatment at the Unit of Orthodontics and Pediatric Dentistry, Section of Dentistry, Department of Clinical, Surgical, Diagnostic and Pediatric Sciences, University of Pavia, Italy.

Sample size calculation (alpha = 0.05; power = 90%) was calculated for two independent study groups concerning the variable skeletal divergence (primary outcome) [13]. The expected difference between the means was supposed to be 2.8; therefore, 100 patients were requested for each group.

The study sample consisted of 200 patients divided into two groups: the experimental group included 100 patients diagnosed with a temporomandibular disorder according to the Diagnostic Criteria for the Temporomandibular Disorders (DC/TMD); the control group consisted of 100 patients with no signs or symptoms of TMD.

The following inclusion criteria were used for the study: (1) patients aged between 18 and 30; (2) recruited from 2014 to 2019; (3) X-ray examinations (orthopantomography and teleradiography) performed at the Department of Orthodontics and Pediatric Dentistry, University of Pavia, Italy; (4) permanent dentition; (5) absence of systemic diseases affecting bone structure; (6) absence of trauma history; and (7) absence of neurological diseases.

Each record was anonymized and consecutively analyzed.

### 2.1. X-Ray Analysis

Panoramic radiography is commonly used to assess the extent of mandibular asymmetry, as bilateral information is provided in routine dental practice [14]. The asymmetry indices of the mandibular height based on the ratio of condylar height (CH) and ramus height (RH) asymmetry, according to Habets' method [15], correlated significantly between TMD and non-TMD patients [16, 17].

A single operator (LB) calculated condylar asymmetry on anonymized orthopantomography (CRANEX™ D, SOREDEX, GE Healthcare Finland Oy) using GIMP software (the GIMP team, GIMP 2.8.10, http://www.gimp.org, 1997-2014, retrieved on 31.07.2014).

The most lateral points of the condyle and the ramus were marked X and Y on the left and right sides.

The lines (ramus tangent) were drawn passing through X and Y (A-line). To the A-lines (the ramus tangent) from the most superior points of the condylar images, perpendicular B-lines were drawn, and the intersection points were called Z points [13].

**Figure 1:**
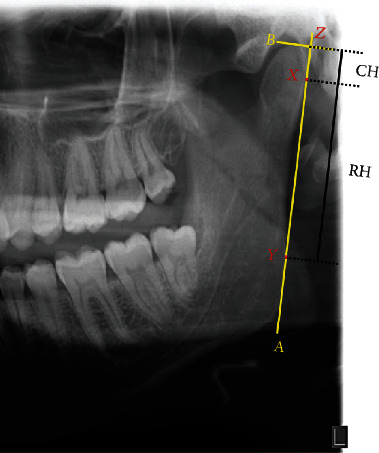
OPG analysis. Measurement of condylar height symmetry: the most lateral point of the condyle (X) and the ramus (Y) are individuated, and a line passing through these points (A) is drawn. Then, a line (B) passing through the most superior point of the condyle and perpendicular to A is traced. Condylar height (CH) is the distance in mm between X and the intersection of A and B (Z).

The distances between X and Z were measured and recorded as condylar height (CH). Similarly, the distances between X and Y and between Z and Y were measured and recorded as ramus height (RH) and condylar plus ramus height (CH+RH), respectively (Figure 1).

The asymmetry indexes of the condyle, the ramus, and the condyle plus ramus were computed by the following formula developed by Habets et al. [15]:

(1) $$\begin{array}{c} \text{Asymmetry index }\left(\text{AI}\right)\text{: }\;|\;\frac{\text{R}-\text{L}}{\text{R}+\text{L}}\;|\;*100. \end{array}$$

Orthopantomography is commonly affected by magnification [18–20]; in this case, the issue is overcome because a rapport is used and not an absolute measurement [21]. According to Habets' studies, values of condylar asymmetry smaller than 6% are not clinically relevant, while values greater than 6% are considered true skeletal asymmetry [22].

**Figure 2:**
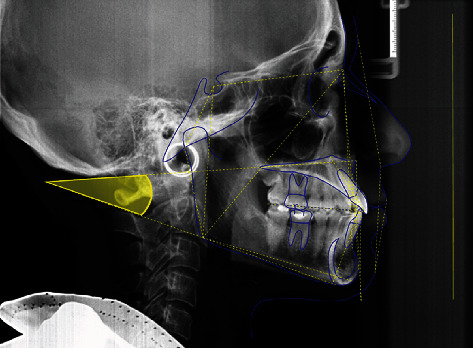
Cephalometric analysis. Measurement of the angle between the plane passing through the anterior and posterior nasal spine (SnaSnp) and the plane passing through the gonion and gnathion mandibular points (GoGn).

Cephalometric analysis was performed via Delta-Dent software 2.2.1 (Outside Format, Spino d'Adda, CR, Italy) by a single skilled operator to measure the angle between the plane passing through the anterior and posterior nasal spine (SnaSnp) and the plane passing through the gonion and gnathion mandibular points (GoGn) (Figure 2). Patients showing a value of SnaSnp^GoGn^ angle greater than 25° have been classified as hyperdivergent; patients with an angle lower than 15° are hypodivergent [18]. The normal value is 20 ± 5°.

### 2.2. Statistical Analysis

Numeric analysis of the data was performed using computer software (R® version 3.1.3, R Development Core Team, R Foundation for Statistical Computing, Wien, Austria). Descriptive statistics including mean, standard deviation, minimum, median, and maximum values were calculated for all numerical groups.

A linear regression model for TMD was performed, adding as covariates the age, sex, symmetry, and divergence.

The significance was predetermined at *p* < 0.05 for all tests.

## 3. Results

200 patients (80 males and 120 females, mean age 22.52 years) was divided into two groups according to the positive or negative diagnosis of TMD following the DC/TMD: the experimental group included 100 patients; the control group consisted of 100 patients with no signs or symptoms of TMD.

Descriptive statistics are reported in Table 1.

Comparing the trial group with the control group, patients with a TMD diagnosis showed significantly greater skeletal divergence with a greater SpP^GoGn^ angle (*p* = 0.00155).

The analysis of the condylar symmetry parameter revealed a strong statistically significant difference between the two groups, with the TMD group having a much higher percentage of asymmetric condyles (*p* < 0.0001).

As for the sex factor, there was a statistically significant difference between the two groups (*p* = 0.0444), while no difference in age was detected (*p* = 0.297).

The summary of the data is given in Table 2.

## 4. Discussion

The null hypothesis of the study was partially rejected. Significant differences between control and TMD groups were reported for some variables.

The present report evaluated orthopantomographical and teleradiogaraphical data. Together with the objective examination and photographs, X-rays are the most suitable method for evaluating the craniofacial complex. There is no doubt that three-dimensional examinations such as CBCT make it possible to have more reliable and precise information and are therefore to be preferred [21, 23]. Even today, however, they cannot be used as routine examinations for an unfavourable cost-benefit ratio, both in terms of economic costs and in terms of radiation dose. 3D examinations are mandatory in cases with severe malformations, which require maxillofacial surgery treatment, but cannot be carried out on all patients. For this reason, bidimensional exams such as orthopantomography and teleradiography can be used to obtain preliminary information.

This study was aimed at evaluating the association between vertical skeletal patterns, condylar height symmetry, and temporomandibular disorders in adults.

The difference between the two groups in terms of gender and age was assessed as a secondary objective.

The results showed a higher number of TMD patients with asymmetrical condyles detected in orthopantomography (Habets' method) [15], a significant difference in skeletal divergence (with the trial group being more hyperdivergent), and a higher females percentage affected by TMD problems.

On the contrary, no differences were found between the two groups in terms of age.

Nowadays, where scientific evidence refutes the correlation between TMD and occlusion, skeletal factors can be inserted into the multifactoriality of these pathologies.

In fact, various studies have shown that there is a relation between the Class II skeletal growth pattern and hyperdivergence with TMD problems [24–27].

**Figure 3:**
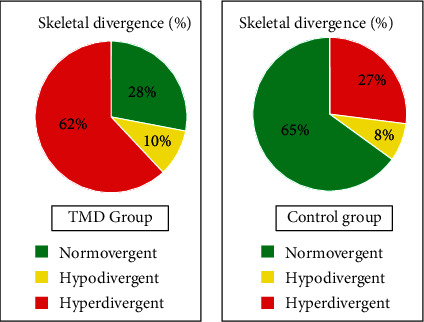
Skeletal divergence: sample distribution.

The sample we analyzed showed a greater presence of subjects with craniomandibular hyperdivergence among TMD patients with a percentage of 62%; on the contrary, the healthy subjects had a mostly normodivergent pattern (64%) (Figure 3).

A systematic evaluation, published in 2015 by Manfredini et al., also confirmed the relation between the hyperdivergence facial growth pattern (HPG) with increased TMJ displacement and degenerative joint disease [12].

A posterior rotation of the jaw associated with a more angled chewing force vector causes an anterior rotation of the condyle; this could probably facilitate the onset of TMD [18].

**Figure 4:**
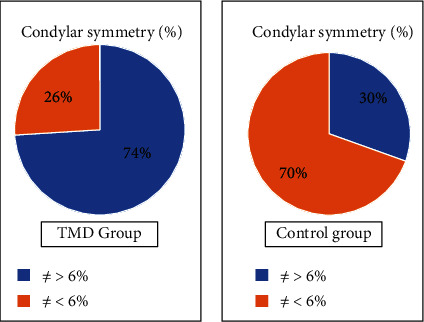
Condylar symmetry: sample distribution.

As for the results of condylar symmetry, detected in orthopantomography, they show an extremely higher percentage, 74%, of asymmetric subjects in the TMD group confirming other previous studies [28, 29], which indicated a correlation between basal asymmetry and increased risk of developing TMD (Figure 4).

Further out, the analysis of the control group sample is also in line with previous studies: Piancino et al. [30] observed that patients with normal occlusion and without temporomandibular joint disorders showed symmetrical condyles measured in orthopantomography, with a condylar symmetry index of 1.72% ± 1.21% [18].

In this study, the percentage of asymmetric cases in the population of the control group stands at 30%.

As a secondary objective, we investigated the correlation between gender, age, and diagnosis of TMD.

Clinical and experimental responses to pain have substantial gender differences, as shown by previous studies [31, 32]. Women have a higher prevalence of painful conditions than men [32, 33]; orofacial pain and other TMD symptoms are often present, with proportions ranging from 2 to 6 women for each man, usually between 20 and 40 years old [34–37].

A hormonal influence could explain the sexual imbalance in the prevalence of TMD [38, 39]. This hypothesis is based on animal and human studies that have suggested that sex hormones may predispose to cartilage rupture and TMJ dysfunction [40, 41].

**Figure 5:**
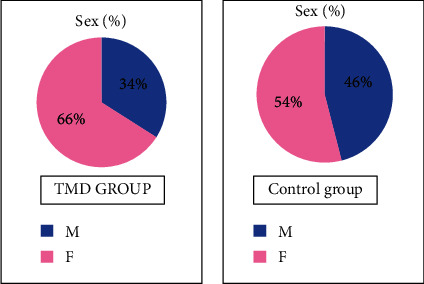
Sex: sample distribution.

This study confirms the results of previous studies: the percentage of female subjects is 66% in the group with DTM, while the control group consists of 54% women and 46% men (Figure 5).

Patients with TMD symptoms present over a broad age range; however, there is a peak occurrence between 20 and 40 years of age [4].

Their prevalence is low in younger children but increases with each age group until they reach maturity. Clinical signs in children are generally mild; serious malfunctions occur only in a small number of cases. Contrary to what could be supported, it has been found that signs and symptoms do not increase with age in adults; in fact, according to studies carried out on elderly subjects, the spread of reported symptoms decreases substantially with age.

The sample chosen for the study is within the typical range of pathology manifestation, which is why there were probably no statistically significant differences in the age distribution of patients between the two groups.

The limitations of this study may be that the sample recruited is represented by a geographically defined population; further studies involving other types of populations would be necessary.

## 5. Conclusion

Although a direct cause-and-effect relationship cannot be demonstrated, since condylar asymmetry and increased skeletal divergence could be consequences of TMD, as evidenced by previous studies [42–44], according to this retrospective study, patients with temporomandibular joint disorders are more likely to show condylar asymmetry and hyperdivergent skeletal pattern. For this reason, preliminary screening like orthopantomography and teleradiography could be useful to intercept patients with functional problems in our clinical practice.

Future studies with orthopantomography and teleradiography images generated from 3-dimensional data could be suggested to overcome the limitations of conventional and bidimensional images. It would also be interesting to monitor the time duration of the data collected by following patients over time to see if the variables change.

## Figures and Tables

**Table 1:** Descriptive statistics.

	Condylar symmetry (%)		Divergence (°)		Age (years)	
	Control	TMD	Control	TMD	Control	TMD
Mean	4.69	10.13	22.84	25.61	22.75	22.30
SD	3.67	5.88	5.33	6.81	3.82	3.90
Min	0.00	0.83	6.80	8.10	18.00	18.00
Mdn	3.90	8.78	23.95	26.65	22.00	21.00
Max	16.13	25.84	34.00	43.00	30.00	30.00

**Table 2:** Demographic and clinical characteristics.

Demographic characteristics				
	Total sample	TMD group	Control group	Significance
*N*	200	100	100	
Age (mean, SD) (years)	22.52	22.75 (3.8)	22.3 (3.9)	*p* = 0.297
Male (%)	40.0	34.0	46.0	*p* = 0.0444
Female (%)	60.0	66.0	54.0	
Clinical characteristics				
	Total sample	TMD group	Control group	Significance
Divergence (°)		*N* (%)	*N* (%)	*p* = 0.00155
Normovergence	93	28.0	65.0	
Hypodivergence	18	10.0	8.0	
Hyperdivergence	89	62.0	27.0	
Condylar symmetry (%)				*p* < 0.0001
Symmetric	96	26.0	70.0	
Asymmetric	104	74.0	30.0	

## Data Availability

Data are available upon request to the corresponding author.
